# Pyoderma gangrenosum after totally implanted central venous access device insertion

**DOI:** 10.1186/1477-7819-6-31

**Published:** 2008-03-06

**Authors:** Ihsan Inan, Patrick O Myers, Rolf Braun, Monica E Hagen, Philippe Morel

**Affiliations:** 1 Visceral Surgery Unit, Department of Surgery, Geneva University Hospital, Rue Micheli-du-Crest 24, CH-1211 Geneva, Switzerland; 2 Dermatology Department, Geneva University Hospital, Rue Micheli-du-Crest 24, CH-1211 Geneva, Switzerland

## Abstract

**Background:**

Pyoderma gangrenosum is an aseptic skin disease. The ulcerative form of pyoderma gangrenosum is characterized by a rapidly progressing painful irregular and undermined bordered necrotic ulcer. The aetiology of pyoderma gangrenosum remains unclear. In about 70% of cases, it is associated with a systemic disorder, most often inflammatory bowel disease, haematological disease or arthritis. In 25–50% of cases, a triggering factor such as recent surgery or trauma is identified. Treatment consists of local and systemic approaches. Systemic steroids are generally used first. If the lesions are refractory, steroids are combined with other immunosuppressive therapy or to antimicrobial agents.

**Case presentation:**

A 90 years old patient with myelodysplastic syndrome, seeking regular transfusions required totally implanted central venous access device (Port-a-Cath^®^) insertion. Fever and inflammatory skin reaction at the site of insertion developed on the seventh post-operative day, requiring the device's explanation. A rapid progression of the skin lesions evolved into a circular skin necrosis. Intravenous steroid treatment stopped the necrosis' progression.

**Conclusion:**

Early diagnosis remains the most important step to the successful treatment of pyoderma gangrenosum.

## Background

Patients undergoing totally implanted central venous access device (TICVAD) insertion are frequently at risk of infection, firstly by implanting foreign material, which can be colonized and difficult to treat, secondly because the underlying disease often is associated with a decreased immune response such as metastatic malignant diseases and haemopathies. The first aetiology of inflammatory ulcerative skin lesions associated with TICVAD insertion is thus usually assumed to be bacterial infection [1]. However, the differential diagnosis of these skin lesions is quite wide, and must be considered in all its breadth when managing such lesions after TICVAD insertion. One can name bacterial (including mycobacterial) skin infections, necrotizing fasciitis, deep mycosis, chronic herpes simplex infection, vasculitis (Wegener's disease), antiphospholipid-antibody syndrome, parasitic infection (cutaneous leishmaniasis or amebiasis), halogene dermatitis, coumarine necrosis or injection drug abuse with secondary infection as most frequent causes of such lesions[2,3]. Pyoderma gangrenosum (PG) is a rare, aseptic skin disease, which should be considered in the differential diagnosis. To our knowledge, we report the first case of PG after TICVAD insertion and discuss the difficulties in management that such cases represent.

## Case presentation

**Figure 1:**
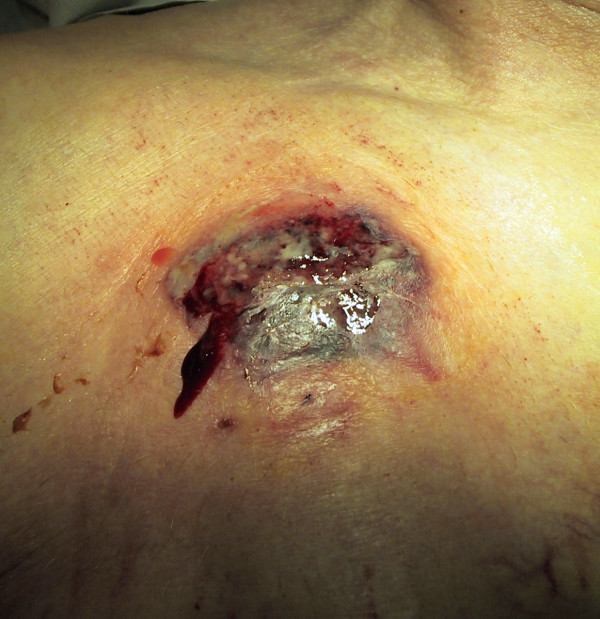
7 days after TICVAD implantation.

**Figure 2:**
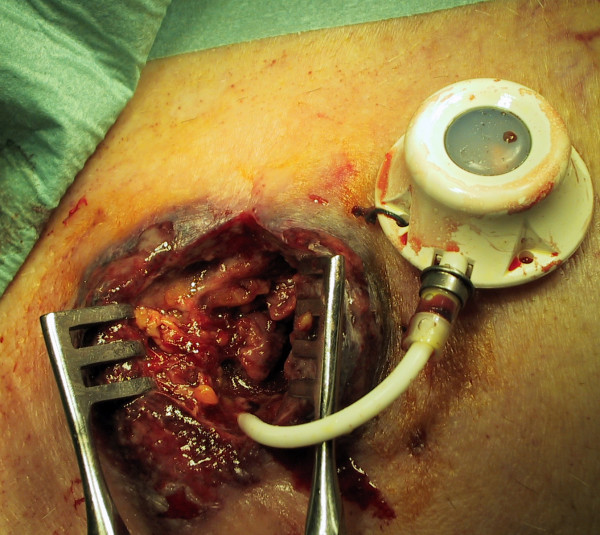
During the extraction of TICVAD.

A 90 year-old patient in good general health, known for a myelodysplastic syndrome with refractory anaemia and myelofibrosis, became transfusion and thrombapheresis dependent, requiring implantation of a right subclavian TICVAD. Thereafter he developed dyspnoea and a fever of 38.6°C, motivating hospitalisation at the 7th postoperative day. An ischemic left cardiac decomposition was diagnosed, in the context of positive troponins, anterolateral ischemic signs on the ECG and severe anaemia (haemoglobin 68 g/l). Important skin inflammation with central necrotic ulceration and violet coloration of the edges was noted on the site of the TICVAD (figure 1 and 2). Laboratory investigations revealed an inflammatory state (leucocytosis at 11,8 G/l, non segmented neutrophils 8%, C-Reactive protein 175 mg/l). The TICVAD was removed on the 8th post-operative day, cultures were taken and wide-spectrum antibiotics (cefepime and vancomycine) were introduced. Because of persistent fever and progressive renal failure, the antibiotics were changed to imipenem and teicoplanine. Cultures showed that pathogenic bacteria are not involved.

**Figure 3:**
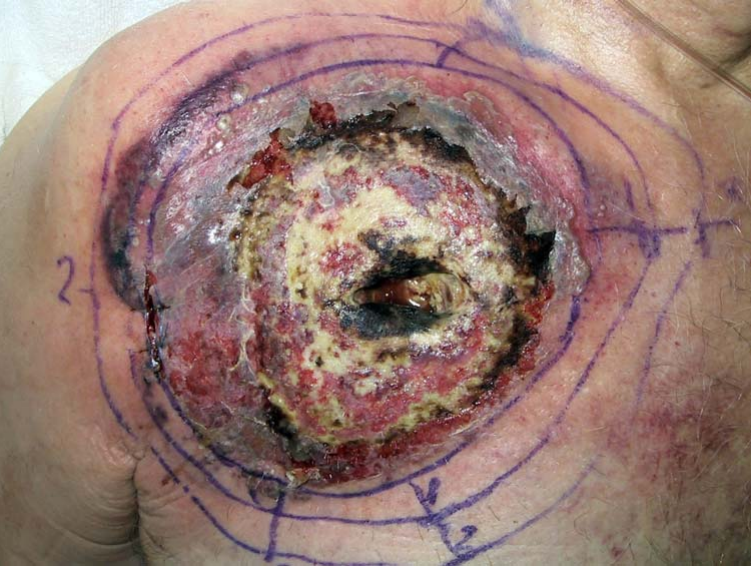
5 days post extraction, diagnostic of PG retained.

Despite these antibiotics, a fever and an inflammatory state persisted. The skin necrosis progressed rapidly around the TICVAD explanation site to the right upper chest wall (figure 3). A biopsy of the necrosis's edge revealed non-specific inflammation, diagnosis of PG is retained on clinical evolution. Corticosteroid therapy was started, improving both the skin lesions and systemic inflammatory signs.

Unfortunately, the patient developed acute anuric renal failure of mixed aetiology (systemic inflammatory response syndrome, toxic to vancomycine and pre-renal). The patient died on the 15th post-operative day.

Of note, the patient was hospitalised in our institution one year earlier for a rapidly growing necrosis, bordered with a violet coloration, of the distal phalanx of the right index finger after a minor trauma. Despite antibiotic treatment and successive debridements and amputations, the last of which was at the metacarpo-carpal joint, the ulcer progressed. Cultures remained sterile. The hand healed 6 months later with conservative treatment. The diagnosis of PG was not evoked at that time.

## Discussion

Pyoderma gangrenosum is an aseptic skin disease. The aetiology of pyoderma gangrenosum is unclear. Pyoderma gangrenosum was first reported in 1924 following drainage of an abdominal abscess [4] and formally described in 1930 [5] as an unusual skin eruption reported in five cases, four of which had chronic ulcerative colitis. It was given such a name because the authors believed that streptococcal infection was a significant component leading to secondary cutaneous gangrene. This was shown not to be relevant, although the cause of PG remains obscure, most probably an immunological anomaly of the hyperergic reaction type. IL-8, a potent leukocyte chemotactic agent, has been shown to be overexpressed in PG ulcers and to induce similar ulceration in human skin xenografts transfected with recombinant human IL-8[6]. IL-16, a neutrophil chemotactic agent, has also been implicated[3,7]. The factors inciting or maintaining these abnormalities are unclear but likely are multiple, mixing genetic predisposition, undefined infectious agents or paraneoplastic or paraimmune phenomena[3].

PG is a diagnosis of exclusion[8]. The distinctive clinical features of PG are apparent enough to permit the diagnosis of most cases[3], comprising in it's classic form a burrowing ulcer with an irregular margin and ragged purple-red overhanging edge. Lesions can be solitary or multiple, chronic or recurrent, most often localized on the legs, particularly in the pretibial region, or, in patients who have undergone colectomy for inflammatory bowel disease (IBD), around a colostomy. The initial lesion starts with an inflammatory papule or follicular pustule, surrounded by erythema or a haemorrhagic bulla with an erythematous base. It evolves into an ulcer with a purulent base and a violaceous border, which progresses outwards. Ulcers heal leaving an atrophic-pigmented and cribriform scar.[3,9]

PG has been classified into four different types: ulcerous, pustulous, vegetans and bullous[3,10] (Table 1).

**Table 1:** Classification of PG

Ulcerous PG	The most common form is the ulcerous type. It is rapidly progressive, severely painful and characterized by a necrolytic, mucopurulent deep ulcer with an undermined, violaceous, oedematous livid border. PG ulcer is a dynamic process, rapidly destroying skin tissue, producing a liquefactive necrosis. It is associated with arthritis (37%), IBD (30%), hematological malignancies, multiple myeloma, paraproteinema and other conditions. Aggressive immunosuppressant treatment is indispensable.
Pustulous PG	Pustulous PG is characterized by multiple lesions with inflammatory borders. It is strongly associated with IBD. Upon remission of IBD, skin lesions also improve.
Vegetans PG	Vegetans PG is solitary, slowly progressive form of PG, characterized by superficial ulcerations with defined borders, sometimes presenting exophytic growth. Less aggressive systemic or topical treatment is usually sufficient.
Bullous PG	Bullous PG is characterized by painful superficial bullae with progressive ulceration and erythematous borders. It is associated with myeloproliferative syndromes and has a poor prognosis if associated with leukemia. Systemic immunosuppression is necessary.

Extracutaneous manifestations are also described, most frequently pulmonary, although all organs can be affected. Some patients develop fever asthenia, myalgias, and arthralgias. PG can lead to multiorgan system failure in the context of severe inflammatory response syndrome. The histopathological features of PG are relatively aspecific, revealing important neutrophilic infiltrate, haemorrhage and necrosis of the epidermis, but are useful in ruling out other causes of ulceration[3,9].

Approximately fifty to 70% of cases are associated with a systemic disease, usually IBD, arthritis or haematological disorders. Both type and severity of associated disease is important on the prognosis of PG. Most frequent haematological disorders associated include acute myeloblastic leukaemia, "hairy cell" leukaemia, myelodisplasic syndrome, myelofibrosis and IgA monoclonal gammopathy. Most of the time successful treatment of associated disease results in remission of PG. Some cytokines such as granulocyte colony-stimulating factor, interferons and antipsychotic drugs have been shown to induce PG.[2,9]

Pathergy, i.e. lesions developing at the site of minor trauma or surgery, is observed in 25–50% of cases[11]. PG has been observed on the incision site after digestive surgery, hernia repair, gynaecologic surgery, breast surgery, plastic surgery and cardiovascular surgery.

Diagnostic exams should include skin biopsy of the border of the skin lesions for histology and culture (including yeast, parasites and mycobacteria), complete blood count (and marrow aspiration/biopsy if pathological), sedimentation rate, c-reactive protein, renal and hepatic function, serum and urinary protein electrophoresis and immunelectrophoresis, anticardiolipid antibodies, VDRL, p- and c-ANCA, cryoglobulines, coagulation, chest x-ray and endoscopic examination of colon and rectum. [9]

The case that we report demonstrates how wide the differential diagnosis of inflammatory ulcerative skin lesions is, and how difficult is to diagnose pyoderma gangrenosum. Skin lesions were attributed to bacterial infection due to recent TICVAD operation. PG was considered because of persistent cultures showing that pathologic bacteria are not involved and absence of lesion improvement under different antibiotics. Despite the non-specific biopsy, the diagnosis of PG retained on clinical basis and treatment was started. Unfortunately, the patient developed such an extensive inflammatory response associated with toxic renal failure that death was inevitable at this stage. The recurrent skin necrosis of the right hand a year prior should retrospectively be attributed to PG. A history of previous ulcerative skin lesions of unknown aetiology resulting in amputation in a patient presenting characteristic ulcerative lesions after TICVAD implantation should make one evoke the diagnosis of PG.

The aim of the therapy is to prevent the progression of the ulcers, encourage reepithelisation and decrease pain[12]. There is no specific or standard therapeutic strategy for PG. Despite the large number of treatment proposed, controlled clinical trials are lacking [2]. Immunosuppression is the basis of treatment; corticosteroids and cyclosporine are the most commonly used drugs. Sequence and combination of topical and systemic treatment is empirical and frequently depends on local experience[10].

Topical treatment is generally insufficient as monotherapy and used as supportive treatment for systemic treatment[12]. Topical treatment includes topical or intralesional corticosteroids, tacrolimus ointment, intralesional cyclosporine, topical 5 aminosalicylic acid, nitrogen mustard or 0,5% nicotine cream[2,13].

Systemic treatment is started in most of the cases with corticosteroids (e.g., methylprednisolone 0.5–1 mg/kg/d) or cyclosporine (e.g., 5 mg/kg/d) alone and considered as first-line therapy. Stabilization of the disease is usually achieved within 24 hours. For cases refractory to first line therapy with concomitant inflammatory bowel disease, second line treatment includes biological response modifiers and immunomodulatory therapy. Tacrolimus, thalidomide, azathioprine, dapsone, mycophenolate mofetil and infliximab are shown to be effective in case reports or small series. In cases without associated disease, intravenous immunoglobulins, granulocyte and monocyte adsorption apheresis plasmapheresis and cyclophosphamide treatment are also reported to be effective.[2]

Debridement or necrosectomy in postoperative PG is contraindicated [11]. Elective surgery for other indications should be deferred, and if unavoidable, it should be performed in conjunction with systemic PG therapy[2].

## Conclusion

PG represents a diagnostic challenge. In the presence of a patient with cutaneous inflammatory and necrotizing lesions one must consider PG as a differential diagnosis. Early diagnosis remains the most important step to the successful treatment of pyoderma gangrenosum

## Competing interests

The author(s) declare that they have no competing interests.

## Authors' contributions

**II **carried out the surgical care of the patient and the follow-up during the treatment, realised the illustration and drafted the manuscript. **POM **participated to manuscript draft and literature research. **RB **participated in the follow-up of the patient, diagnosis of the disease and treatment as well as manuscript draft on dermatologic aspect. **MEH **participated to manuscript draft and literature research. **PM **encouraged the case report, participated in its preparation and helped to draft the manuscript. All authors read and approved the final manuscript.
